# Skeletal muscle phenotyping of Hippo gene-mutated mice reveals that *Lats1* deletion increases the percentage of type I muscle fibers

**DOI:** 10.1007/s11248-021-00293-4

**Published:** 2022-01-05

**Authors:** Fakhreddin Yaghoob Nezhad, Annett Riermeier, Martin Schönfelder, Lore Becker, Martin Hrabĕ de Angelis, Henning Wackerhage

**Affiliations:** 1 Exercise Biology Group, Faculty of Sport and Health Sciences, Technical University of Munich, Munich, Germany; 2 German Mouse Clinic, Institute of Experimental Genetics, Helmholtz Zentrum München, Neuherberg, Germany; 3 Chair of Experimental Genetics, TUM School of Life Sciences, Technische Universität München, Freising, Germany; 4 German Center for Diabetes Research (DZD), Neuherberg, Germany

**Keywords:** Hippo pathway, Skeletal muscle, Transgenic mice, Lats1, Fiber type

## Abstract

The Hippo signal transduction network regulates transcription through Yap/Taz-Tead1-4 in many tissues including skeletal muscle. Whilst transgenic mice have been generated for many Hippo genes, the resultant skeletal muscle phenotypes were not always characterized. Here, we aimed to phenotype the hindlimb muscles of Hippo gene-mutated *Lats1*^−/−^, *Mst2*^−/−^, *Vgll3*^−/−^, and *Vgll4*^*+/−*^ mice. This analysis revealed that *Lats1*^−/−^ mice have 11% more slow type I fibers than age and sex-matched wild-type controls. Moreover, the mRNA expression of slow *Myh7* increased by 50%, and the concentration of type I myosin heavy chain is 80% higher in *Lats1*^*−/−*^ mice than in age and sex-matched wild-type controls. Second, to find out whether exercise-related stimuli affect Lats1, we stimulated C2C12 myotubes with the hypertrophy agent clenbuterol or the energy stress agent AICAR. We found that both stimulated *Lats1* expression by 1.2 and 1.3 fold respectively. Third, we re-analyzed published datasets and found that *Lats1* mRNA in muscle is 63% higher in muscular dystrophy, increases by 17–77% after cardiotoxin-induced muscle injury, by 41–71% in muscles during overload-induced hypertrophy, and by 19–21% after endurance exercise when compared to respective controls. To conclude, *Lats1* contributes to the regulation of muscle fiber type proportions, and its expression is regulated by physiological and pathological situations in skeletal muscle.

**Supplementary Information:**

The online version contains supplementary material available at 10.1007/s11248-021-00293-4.

## Introduction

We use our ≈650 human skeletal muscles to move, stand, speak, generate heat, store glucose as glycogen, and amino acids as protein. Skeletal muscles also vary greatly within and in-between individuals. For example, muscle contributes from 20 to 50% to total human body mass (Janssen et al. 2000) and this is due to a more than twofold variation of mean fiber cross-sectional areas and fiber numbers (Lexell et al. 1988). In addition, genetics and years of training can increase muscle mass and function in athletes (Sarzynski and Bouchard 2020) whereas aging, disability, immobilization, and diseases such as muscular dystrophy, cancer, and diabetes impair or reduce muscle metabolism, function, and size (Bonaldo and Sandri 2013; Wolfe 2006).

Muscle size and function are regulated by many signaling pathways and depend on genetics. In the past two decades, the Hippo signal transduction network has been identified as a regulator of skeletal muscle development, regeneration, and size and is associated with diseases such as muscular dystrophy and rhabdomyosarcoma (Wackerhage et al. 2014). In mammals, the canonical Hippo pathway consists primarily of the kinases MST1, MST2, LATS1, and LATS2 (Gabriel et al. 2016). Activated LATS1/2 inhibits the transcriptional co-factors YAP/TAZ by phosphorylation of multiple serines (Liang et al. 2014; Zhao et al. 2007). In contrast, unphosphorylated YAP/TAZ can translocate to the nucleus to co-activate TEAD-1–4 transcription factors to initiate the transcription of target genes such as *ANKRD1, CTGF, and CYR61* (Dong et al. 2007; Liu et al. 2010; Oh and Irvine 2008; Ren et al. 2010; Zhao et al. 2010). Finally, VGLL1–4 proteins can also bind TEAD1–4 (Zhou et al. 2016) and may compete for common binding interfaces with Yap and Taz (Figeac et al. 2019; Hori et al. 2020; Koontz et al. 2013; Yamaguchi 2020).

Hippo researchers and the International Mouse Phenotyping Consortium (IMPC; https://www.mousephenotype.org/) have generated Hippo mutant mice for almost all Hippo-related genes. These mice have all been generally phenotyped but a specific, quantitative phenotyping of skeletal muscle is rarely performed and often muscles are not phenotyped at all.

The aim of this study was therefore to phenotype hindlimb muscles of *Lats1*^−/−^, *Mst2*^−/−^, *Vgll3*^−/−^, and *Vgll4*^*+/−*^ mutated mice and to compare these transgenic mice to age and sex-matched wild-type controls.

## Materials and methods

### Ethical approval and husbandry

All animal procedures including ethical statements and housing & husbandry conditions are available on the IMPC portal website (http://www.mousephenotype.org/about-impc/arrive-guidelines).

### Mouse generation and phenotyping

To study the role of the Hippo related genes in skeletal muscle, the hindlimbs of global knockout mice include *Lats1*^*−/−*^ (*Lats1*^em1(IMPC)H^), *Mst2*^*−/−*^ (*Stk3*^em1(IMPC)H^), *Vgll3*^*−/−*^ (*Vgll3*^em1(IMPC)H^), and *Vgll4*^*+/−*^ (*Vgll4*^tm1b(EUCOMM)Hmgu^) were kindly supplied by the Helmholtz Zentrum München (HMGU) in Germany, and the Medical Research Council Harwell (MRC Harwell) in the UK. Mice were generated on a C57BL/6 N background and phenotyping data were collected at the age of 16 weeks. For each of the Hippo-related genes knockout lines, at least three hind limbs of male mice were collected as well as age and sex-matched control mice of equivalent genetic backgrounds and sent to the Technical University of Munich for hindlimb muscle phenotyping. Information on the transgenesis, phenotyping, and data storage is available on the IMPC portal:

#### https://www.mousephenotype.org/understand/the-data/allele-design/

#### https://www.mousephenotype.org/understand/the-data/phenotyping-process%20impress/

#### http://www.mousephenotype.org/

To phenotype the hindlimb muscles of Hippo gene-mutated mice and their matched control, we first dissected the hindlimb muscles of mutated and control mice. The analyzed muscles are the tibialis anterior (TA), extensor digitorum longus (EDL), gastrocnemius, and soleus. After dissection, we weighed the hindlimb muscles and then calculated the relative muscle weight (muscle weight/whole body weight).

### Mouse genotyping

Each *Lats1*^−/−^ and wild-type control mouse was genotyped using mouse skeletal muscle DNA. To isolate DNA, the EDL muscles were digested in lysis buffer (350 µl of Sodium hydroxide (NaOH 50 mM), and 10 µl proteinase K per sample). Following overnight incubation at 55 °C, 120 µl of 5 M NaCl was added and centrifuged for 15 min, and resulting supernatant (350 µl) was added to 250 µl of isopropanol (Carl Roth, Germany). After vortexing for 2 min, centrifugation at 13,000 rpm for 10 min, and 2 h incubation at room temperature (RT), the aqueous phase was collected. DNA precipitation was obtained by addition of 500 µl of 70% Ethanol, short vortexing, and 10 min centrifugation at 13,000 rpm at 4 °C. Finally, the DNA pellets were resuspended in 50 µl ddH2O and were quantitated on a Nanodrop spectrophotometer (ND-100 spectrophotometer; Nanodrop Technologies, Wilmington, DE, USA). For this genotyping study, DNA was amplified using a TAQ-DNA-Polymerase kit (PeqGold VWR, product number 01–1020) as specified by the manufacturer by a PCR master cycler (Eppendorf, Germany). The amplification cycles consisted of 4 min at 95 °C followed by 30 cycles at 95 °C for 30 s, 58 °C for 30 s, and 72 °C for 2.5 min. The primer sequences were as follows: *Lats1*: forward (F) 5′- TCCGGGCAGGACTGATATAC -3′; reverse (R) 5′- TATTACAGGAAATGCTGAATAACTG -3′. The amplification of *Lats1* knock-out and wild-type gene fragments results in 202-bp and 2071-bp products, respectively. An agarose gel electrophoresis of the PCR products is shown in Fig. S3 (see supplementary data).

### Cell culture

Mouse C2C12 myoblasts cells were cultured in Dulbecco’s modified Eagle’s medium (DMEM) (Gibco, Cat#31,885, Waltham, MA, USA), supplemented with 10% fetal bovine serum (Sigma, Germany), and incubated at 37 °C in humidified air with 5% CO_2_. To induce differentiation, the myoblasts were cultured in a growth medium until confluence, then the medium was switched to DMEM with 2% horse serum (Sigma, Germany). The proliferation and differentiation medium was refreshed daily up to 96 h. After 48–96 h of differentiation, C2C12 Myotubes untreated (DMSO) or 24 h treated with exercise stimulates including 100 μM Clenbuterol (Sigma, Germany) to induce hypertrophy, or 1 mM 5-aminoimidazole-4-carboxamide-1-β-D-ribofuranoside (AICAR; Cell Signaling Technology, USA) to induce energy stress.

### RNA isolation, reverse transcription, and quantitative real-time PCR

Following dissection, the soleus muscle samples were homogenized in 1 ml of QIAzol (QIAGEN, Germany) using a Precellys lysing kit (Bertin Instruments, USA) and Precellys homogenizer machine (Bertin Technologies, USA) with high speed for 40 s. C2C12 myotubes were first washed with phosphate-buffered saline (PBS) and then lysed in lysis T buffer (Peqlab Biotechnology GmbH, reference number 12-6634-01) and stored at -80^◦^C. Total RNA was extracted with the peqGOLD Total RNA Kit C-Line (Peqlab Biotechnology GmbH, reference number: 12-6634-01) according to the manufacturer’s instructions. RNA concentrations were determined by a Nanodrop spectrophotometer (ND-100 spectrophotometer; Nanodrop Technologies, Wilmington, DE, USA). RNA purity was ensured by a 260 / 280 ratio (range 2.00–2.11, mean 2.04). For mRNA analysis, cDNA was synthesized using 2 μg of total RNA and qScript XLT cDNA SuperMix (Quantabio, product number 030256) as specified by the manufacturer. cDNA was amplified with a PerfeCTa qPCR fluorescent SYBR Green SuperMix (Quantabio, product number: 023916) using real-time quantitative PCR (Rotor-Gene RG 6000—QIAGEN). The primer sequences used for the gene expression were as follows:

*Lats1*: forward (F) 5′- AATGAAATGATGCGGGTTGGA-3′; reverse (R) 5′- CAGACTTCACCAAACGCTCC-3′;

*Rpl7*: (F) 5′-ACGGTGGAGCCTTATGTGAC-3′ and (R) 5′-TCCGTCAGAGGGACTGTCTT-3′.

We calculated the expression of *Lats1* using the 2^−ΔΔCt^ method (Livak and Schmittgen 2001) and calculated it relative to the *Rpl7* housekeeping gene (Thomas et al. 2014).

### Western blotting

To extract total protein, we homogenized soleus muscles in lysis buffer (0.1% SDS,0,5 M sodium orthovanadate, 0.5% sodium deoxycholate, 50 mM NaF, 1 mM EDTA,150 mM NaCl, 1%Triton-X 100) supplemented with protease and phosphatase inhibitor cocktail (Peqlab Biotechnology GmbH, Germany) using a Precellys lysing kit (Bertin Instruments, USA) and a homogenizer machine (Bertin Technologies, USA) with high speed for 40 s. After centrifugation at 12,000 g for 10 min at 4 °C, the supernatant was transferred to a new tube and assayed to determine the protein concentration of each sample using the Bradford protein assay kit (BioRad Laboratories GmbH, Cat #5,000,111, Munich, Germany). Homogenates of each soleus sample were diluted to a protein concentration of 1 mg/ml using 4 × SDS sample buffer (0.5 M Tris–HCl pH 6.8, 10% glycerol, 2% sodium dodecyl sulfate, 20% β-mercaptoethanol, and 0.05% bromophenol blue). Samples were then heated at 95^◦^C for 5 min, and 30 µg of whole protein lysates were separated by electrophoresis in a 10% SDS-PAGE gel (Bio-rad, Germany). Proteins were then transferred to polyvinylidene difluoride (PVDF) membranes (Bio-rad, Germany) using a Trans-Blot Turbo Blotting System (Bio-Rad, Germany). Following a blocking step (5% non-fat milk powder, 1X Tris-buffered saline,1% Tween-20), the PVDF membrane was incubated overnight with primary antibody and probed with horseradish peroxidase(HRP)-linked secondary antibodies for 1 h at room temperature. The membranes were developed with enhanced chemiluminescence (ECL) (Bio-Rad, Germany), and the signals were detected by an INTAS Chemocam Imager (Royal Biotech GmbH, Germany). The antibodies used in this study were as follows: Anti MyHC-l (1:1000, DSHB Cat# BA-D5, RRID: AB_2235587), Anti-mouse IgG, HRP-linked Antibody (1:2500, Cell Signaling Technology Cat# 7076, RRID: AB_330924). Quantification of immunoreactive bands was performed using the ImageJ software (http://rsb.info.nih.gov/ij/index.html) and the largest band of a Ponceau S stain of the membrane was used for normalization (Romero-Calvo et al. 2010).

### Muscle cryosectioning and histology

The tibialis anterior (TA) and soleus muscles of mice were dissected, frozen in isopentane (2-methyl butane) cooled to near freezing with liquid nitrogen, and sectioned in a cryostat (LEICA CM3050 S) and mounted on SuperFrost® slides (VWR International GmbH, Germany Cat#631–1349) air-dried and stored at -80^◦^C until further analysis.

### Haematoxylin & eosin stain & NADH tetrazolium reductase histochemical stain

To find out whether the mutation of Hippo genes causes abnormalities such as central nuclei, infiltrations or fibrosis, we stained all muscles with hematoxylin and eosin (H&E) using a standard staining protocol. To stain highly oxidative (typically corresponding to type I and IIa fibers) and less oxidative fibers (corresponding to type IIx and IIb fibers), we used an NADH tetrazolium reductase (NADH-TR) histochemical reaction. For this, frozen TA muscle cryosections (10 μm) were allow to thaw at room temperature and incubated with NADH-TR staining solution (2.52 g Tris HCl, 0.10 g Nitro blue tetrazolium, 0.68 g Tris base, dissolved in 100 ml distilled H_2_O & ≈1 mg of NADH added to 1 ml staining solution (pH 7.4) before staining) for 30–60 min in a humidified chamber at room temperature until there was maximal contrast in staining intensity in-between fibers. Then, samples were washed in distilled water.

### Adenosine triphosphatase (ATPase) histochemical stain

To differentiate in-between type I and type II muscle fibers we used an ATPase histochemical reaction with acid preincubation to selectively stain type I fibers brown-black. For this, 10 μm soleus sections were thawed at room temperature for 5 min and pre-incubated in an acetate buffer at pH 4.47 for 10 min followed by an Adenosine 5’-triphosphate reaction solution for 30 min at 37 ^◦^C then rinsing in distilled water. The slides were then incubated in 1% calcium chloride for 3 min then rinsed in distilled water and placed in 2% cobalt chloride solution for 3 min. Following further washes in distilled water, slides were finally incubated in a 1% ammonium sulfide solution for 1 min and washed 3 times in distilled water.

### Dehydration and Mounting

All sections from H&E staining, NADH-TR, and ATPase enzyme activity analysis were dehydrated by sequential dipping in 70% and 100% ethanol and then ROTI Histol (Carl Roth, Germany), before being mounted with coverslips using DPX mounting medium.

### Microscopy

Digital photos of cross-sections were captured in 2.5, 5, and 20-X magnifications using a Zeiss Axio Lab.A1 equipped with a digital camera (Zeiss, Germany) and Zeiss ZEN Software version 2.6 (Blue edition; Zeiss, Germany). To determine the cross-sectional area (CSA), Twenty-five myofibers per fiber type, per sample were analyzed. To distinguish fiber types (type I and II) percentages of muscle fiber types were quantified manually by counting stained and unstained fibers (fivefold magnification). All microscopy analysis was performed blinded using the ImageJ software (http://rsb.info.nih.gov/ij/index.html;RRID: SCR_003070).

### Re-analyses of published datasets

To investigate whether diseases, exercise, or other factors affect *Lats1* expression we retrieved gene expression datasets from Gene Expression Omnibus (GEO). These include dataset (GDS4924) for the regeneration after cardiotoxin-induced muscle injury in TA muscle of mice (Lukjanenko et al. 2013), the transcriptome dataset (GSE23244) for comparison fiber type I and llb in mouse muscle (Chemello et al. 2011), microarray dataset GDS4932 for muscle hypertrophy after synergist ablation-overloaded in plantaris muscle of mice (Chaillou et al. 2013), and microarray dataset (GDS609) for muscle dystrophy in quadriceps muscle of Duchenne Muscular Dystrophy (DMD) patients and unaffected controls (see the supplementary data S1A in Haslett et al. (Haslett et al. 2003). To obtain data on the effects of exercise on *Lats1* expression, we downloaded supplementary data files from studies comparing resting muscle biopsies with biopsies taken at 2.5 h and 5 h after a bout of either endurance or resistance (strength) exercise (Vissing and Schjerling 2014). Lastly, to obtain a forest plot of the expression of *Lats1* gene in response to acute aerobic and acute resistance exercise we re-analyzed the meta-analysis of all available human skeletal muscle studies. The original data was obtained from Meta MEx Portal (http://www.metamex.eu/) (Pillon et al. 2020).

### Statistical analysis

To analyze the normal distribution of data we performed a Kolmogorov–Smirnov (K-S) normality test and then performed an unpaired t-test to test for differences between conditions. Results are expressed as mean ± standard error (SEM) and considered significant at *P* < 0.05. All statistical analyses were performed using Prism version 8.0 statistical software package (GraphPad Prism; RRID: SCR_002798).

## Results

### Effect of Hippo gene mutations on the absolute and relative size of hindlimb muscles

**Fig. 1:**
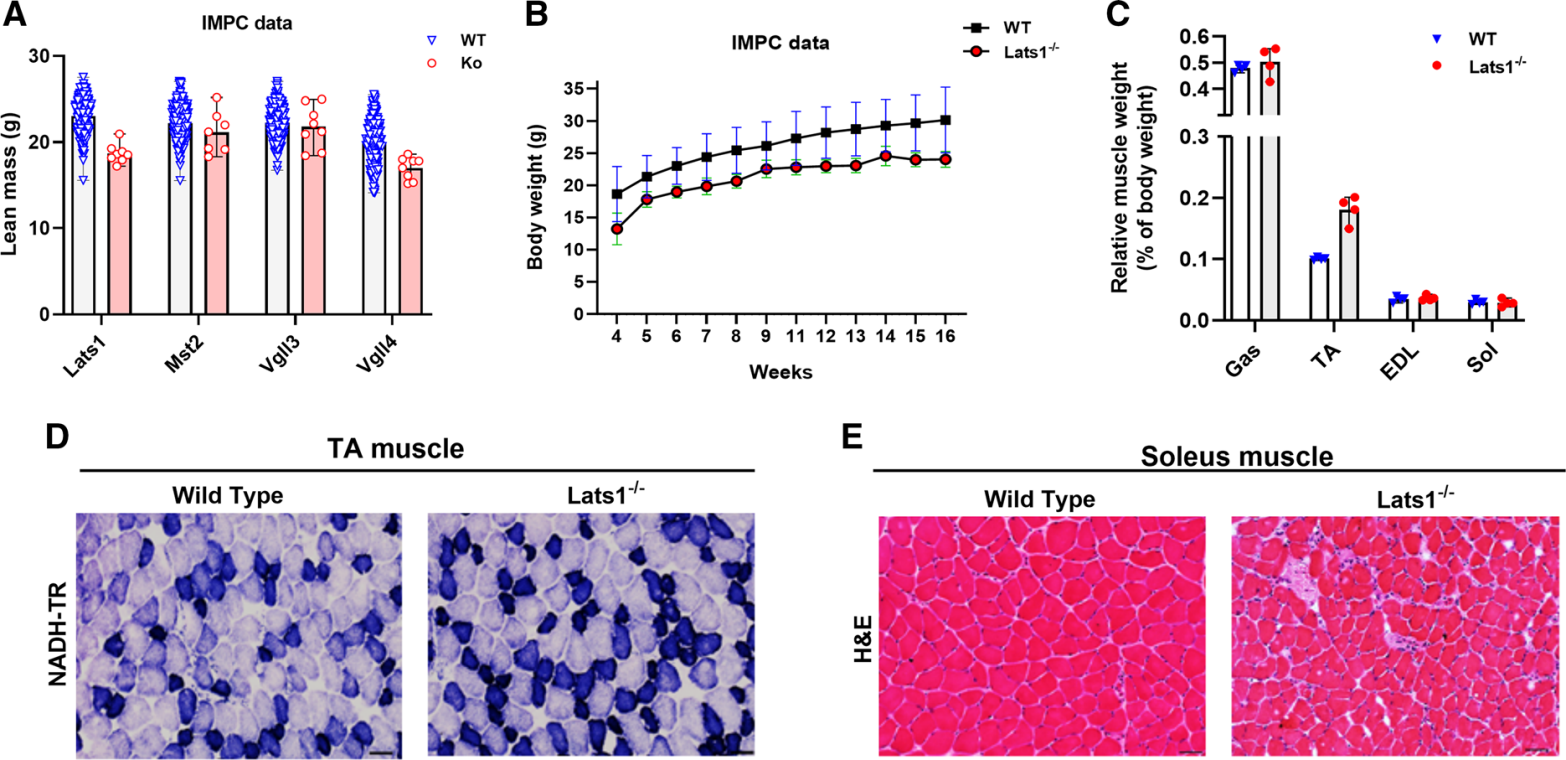
(**a**) Lean mass of Hippo gene-mutated (n = 6–8), and control (n = 499–1850) 16-week-old mice. (**b**) Average of total body weight of *Lats1* knockout (n = 8) and its control (n = 3349) mice. (**c**) Relative muscle weights of Gastrocnemius (Gas), Tibialis Anterior (TA), Extensor Digitorum Longus (EDL), and Soleus (Sol) muscles normalized to total body weight of *Lats1*^−/−^ mice versus wildtype control mice (n = 4). (**d**) NADH-Tetrazolium Reductase (NADH-TR) staining of tibialis anterior muscle cross-sections from control and *Lats1*^*−/−*^ mice (high oxidative capacity is stained in dark blue, low oxidative capacity is stained in light blue; n = 4). (**e**) Hematoxylin and Eosin (H&E) staining of the soleus muscle cross-sections from control and *Lats1*^−/−^ mice (n = 4). Scale Bar = 50 μm. All values are presened as mean ± SEM. *P < 0.05. KO: Knock Out; WT: Wild Type; g: gram; mg: milligram. See Figure S1 for further H&E and NADH staining

To gain information on general phenotypes of Hippo transgenic mice, we first summarised publicly available IMPC phenotype data in Supplemental Table S1. Key phenotypes of Hippo gene-mutated mice were a 52% (*P* < 1.91 × 10^–06^) higher fat mass in *Tead1*^/–^ female mice, a 33% (*P* = 1.64 × 10^–06^) higher grip strength in *Lats1*^−/−^ female mice, and 18.6% (*P* = 1.69 × 10^–08^) less lean body mass in *Lats1*^−/−^ male mice (Fig. 1a). Moreover grip strength was 27% (*P* = 2.92 × 10^–06^) lower in *Lats1*^/–^ male mice when compared to sex- and age-matched wild-type mice (Supplemental table. S1).

Next, to study the effect of transgenesis on skeletal muscle phenotype, we phenotyped hindlimb muscles of *Lats1*^*−/−*^, *Mst2*^*−/−*^, *Vgll3*^*−/−*^, and *Vgll4*^*+/−*^ mice versus sex- and age-matched wild-type mice as controls. The hindlimb of these mice were available from our collaborators (see Acknowledgments). *Lats1*^*−/−*^ muscles were significantly lighter than those of wild-type controls (Fig. 1b) but this is explained by a proportionally lower body weight so that relative muscle weight was unchanged (Fig. 1c). The lower body weight of *Lats1*^*−/−*^ mice is probably explained by the lower growth hormone levels reported in an earlier study of *Lats1* transgenic mice (St John et al. 1999). When inspecting H&E stains of all muscles, we did not detect pathological changes such as central nuclei or cores in any of the mutant mice (Fig. 1d, e; Fig. S1).

### *Lats1* deletion alters muscle fiber type distribution

**Fig. 2:**
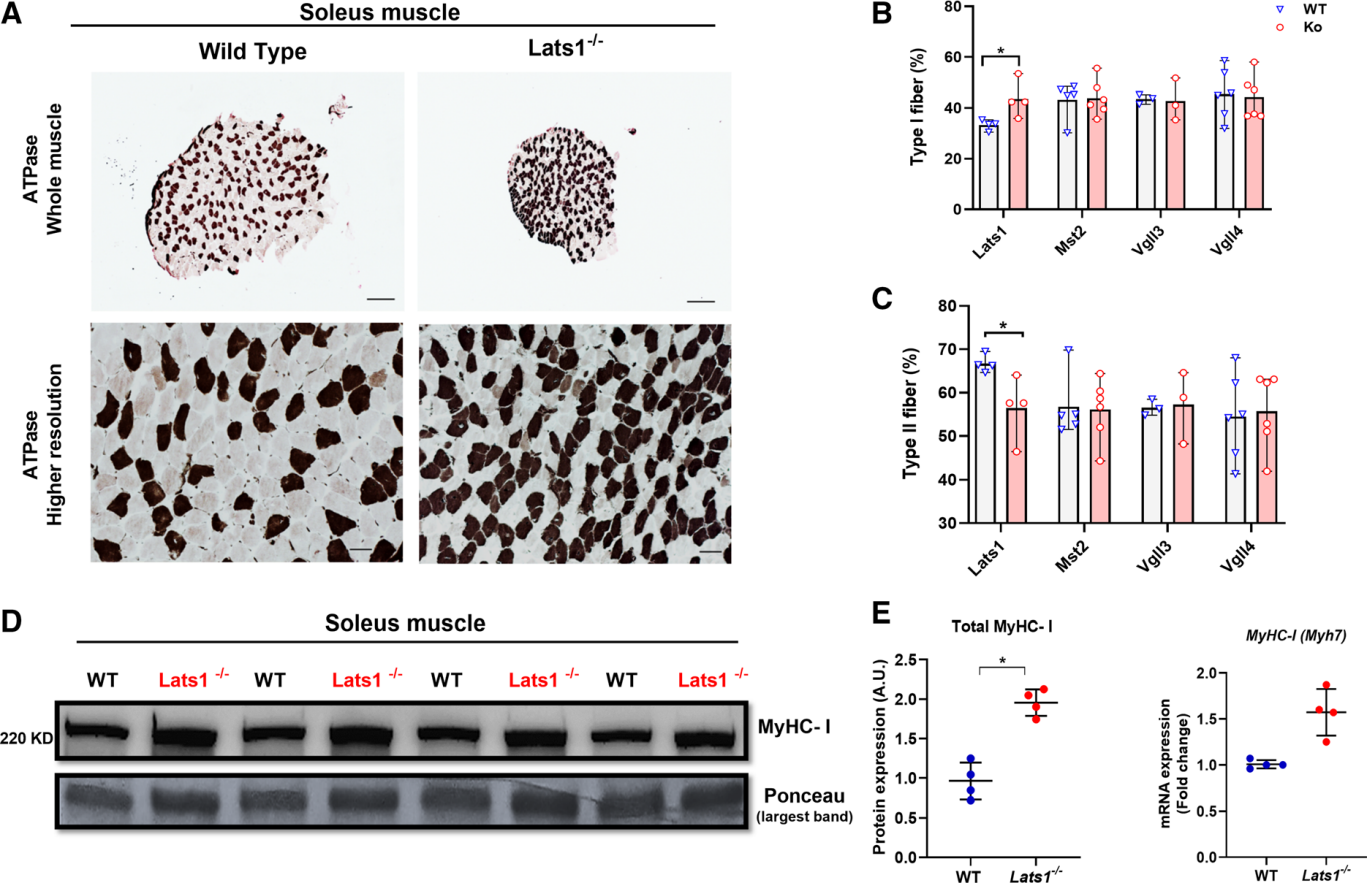
*Lats1* deletion increases slow type I MyHC. (**a**) Representative images of ATPase stained soleus muscles cross-sections from 16-week-old control and *Lats1*^−/−^ mice (n = 4) to determine fiber types (Type I fibers stain dark, type II and IIa fibers stain light). (**b**) Type I and (**c**) IIa fibre percentages in soleus muscles (n = 3–6). (**d**) Protein levels of total MyHC-I in the soleus muscle from control and *Lats1*^−/−^ mice (n = 4). (**e**) Expression levels of *Myh* mRNA (encoding MyHC-I protein) in the soleus muscles from control and *Lats1*^−/−^ mice were measured by qPCR (n = 4). Protein is normalized to the largest band in the Ponceau stain. *Rpl7* was used as a reference gene to normalize mRNA. Circles indicate individual data points. A.U, arbitrary units; KD, Kilo Dalton. Scale Bar = 200 μm (whole muscle); 50 μm (higher resolution). All values present mean ± SEM. *P < 0.05. See Fig. S2 for further ATPase staining figures

Next, we quantified the number and size of type I and IIa muscle fibers in the soleus as it is possible to count all fibers in this muscle. To do so we stained muscle fibers using an ATPase reaction with a pH  4.47 pre-incubation which stains type I fibers black and type IIa fibers white/grey (Fig. 2a). This revealed that *Lats1*^−/−^ mice had 11% more type I fibers and 11% fewer type IIa fibers than age and sex-matched wild-type controls (*p* < 0.05; Fig. 2b–c). In contrast, the muscles of other Hippo-pathway-mutated mice did not differ from matched wild-type controls (Fig. 2b-c and Fig. S2). Consistent with this, in soleus the concentration of slow type I myosin heavy chain (MyHC-l) was 80% higher in *Lats1*^−/−^ mice than in wild-type control mice (*P* < 0.05) (Fig. 2d). Also, *Myh7* mRNA was 50% higher (*P* = 0.067) in the soleus of *Lats1*^−/−^ mice than in wild-type controls (Fig. 2e).

### Effect of exercise-mimicking compounds on *Lats1* expression in vitro

**Fig. 3:**
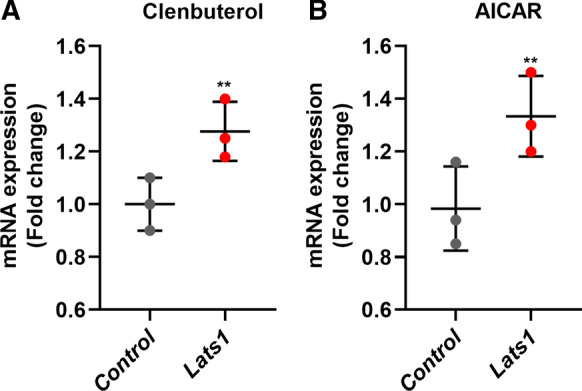
Effect of exercise-associated stimuli on the expression level of the *Lats1* gene in vitro. C2C12 myotubes were incubated with (**a**) Clenbuterol (100 μM), or (**b**) AICAR (1 mM) for 24 h and analyzed for *Lats1* gene expression by qRT-PCR. *Rpl7* was used as a reference gene to normalize mRNA. The circles indicate individual data points. Data are presented as mean ± SEM. **P < 0.001

To investigate whether *Lats1* is regulated by stimuli associated with exercise, we incubated C2C12 myotubes with the energy stress-inducing AMPK-activating drug AICAR and the hypertrophy-inducing β2-agonist Clenbuterol. We then measured *Lats1* gene expression (Fig. 3a–b).

After 24 h treatment with 100 μM of clenbuterol, the expression levels of *Lats1* increased 1.2 fold (Fig. 3a) when compared to control. After 24 h of treatment with 1 mM of AICAR *Lats1* gene expression increased 1.3 fold when compared to control (Fig. 3b) suggesting that exercise-related stimuli modulate *Lats1* expression.

### Bioinformatic analyses of *Lats1* gene expression in skeletal muscle

**Fig. 4:**
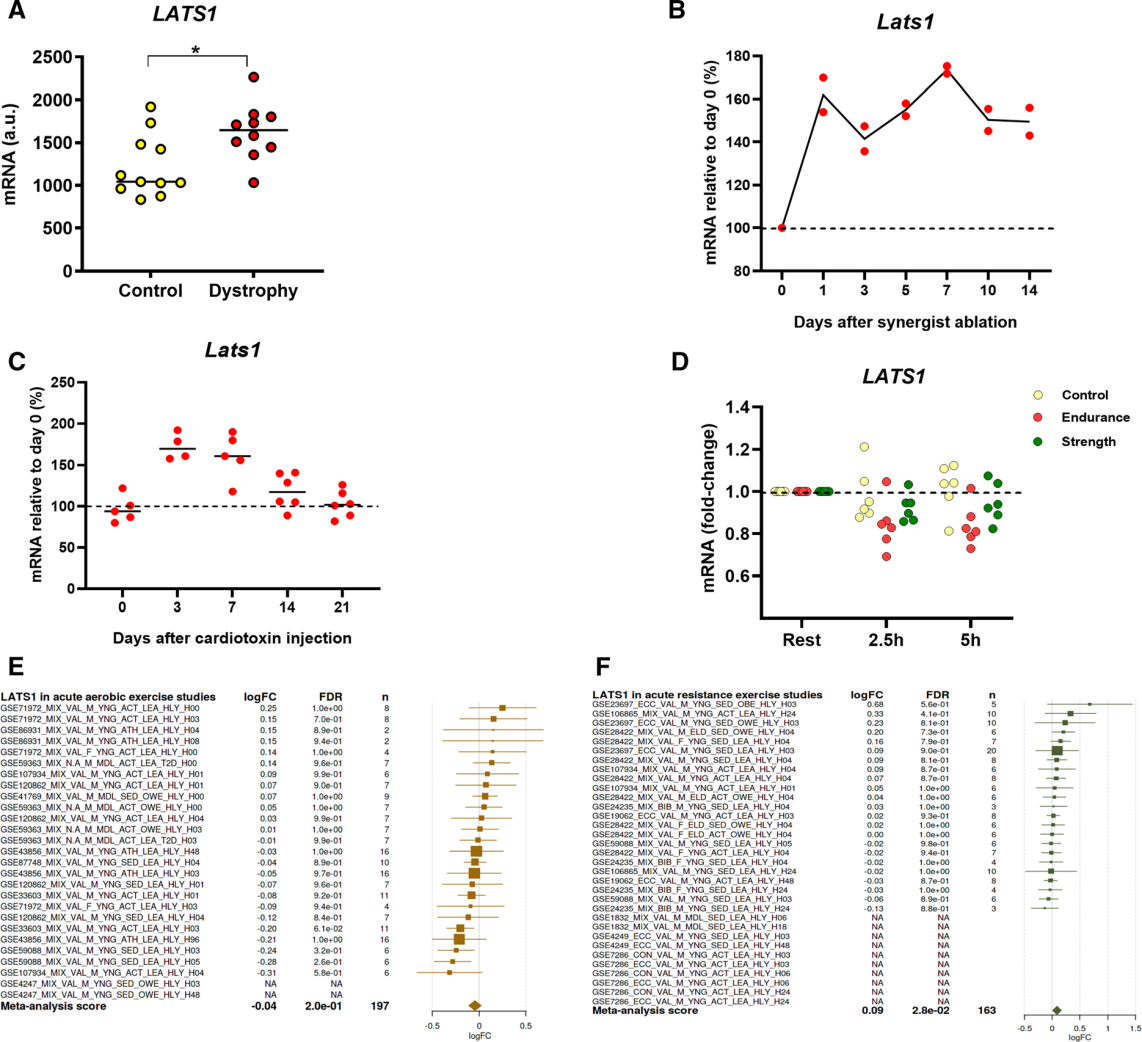
*Lats1* gene expression in response to physiological and pathological factors in skeletal muscle. (**a**) *LATS1* expression in quadriceps muscle biopsy samples of healthy and DMD patients (Haslett et al., 2003). (**b**) Relative mRNA expression of *Lats1* in synergist ablation-overloaded mouse plantaris muscle (Chaillou et al 2013). (**c**) *Lats1* gene expression in mice tibialis anterior muscle injured with cardiotoxin injection at day 0 up to day 21 (Lukjanenko et al. 2013). (**d**) Effect of human endurance and resistance (strength) exercise on the expression of *LATS1* in the vastus lateralis 2.5 h and 5 h after exercise (Vissing and Schjerling, 2014). The circles indicate individual data points. (**e–f**) Meta-analysis of *Lats1 *expression in response to (**e**) acute aerobic and (**f**) acute resistance exercise (http://www.metamex.eu/) (Pillon et al., 2020). Muscle Biopsies were collected at 0 h up to 96 h after exercise. The fold-change (log2) is represented by a square and the 95% confidence intervals are represented by horizontal lines. LogFC, Fold-change (log2); FDR, false discovery rate; n, sample size; A.U, arbitrary units. *P < 0.05

To better understand the regulation of *Lats1* expression and phosphorylation in skeletal muscle we retrieved published datasets from Gene Omnibus or downloaded supplemental data from published papers. This revealed that *Lats1* is more expressed in slow type I muscle fibers in mice than in fast type II fibers (Chemello et al. 2011). The reanalyzed data from Haslett et al. (see Supplementary Data S1A in Haslett et al. 2003) also revealed that mean *LATS1* expression is 63% higher (*P* = 0.014) in the quadriceps of boys with Duchenne Muscular Dystrophy (DMD) when compared to normal quadriceps muscle (Haslett et al. 2003) (Fig. 4a). Moreover, *Lats1* expression increases in regenerating tibialis anterior muscles by 17–77% (Lukjanenko et al. 2013) and in hypertrophying plantaris muscle by 41–71% (See the microarray dataset GSE47098 data (Chaillou et al. 2013)) (Fig. 4b–c), suggesting that *Lats1* is transcriptionally regulated in muscle. In human vastus lateralis, mean *LATS1* expression declines by 19% at 2.5 h and 21% at 5 h (21%) after endurance exercise (Fig. 4d) (Vissing and Schjerling 2014). However, a MetaMex analysis (Pillon et al. 2020) revealed no significant expression changes of *LATS1* in different human skeletal muscles after acute or chronic endurance or resistance exercise (Fig. 4e-f), respectively.

## Discussion

In this study, we compared the hindlimb muscles of *Lats1*^*−/−*^*, Mst2*^*−/−*^*, Vgll3*^*−/−*^*,* and *Vgll4*^*+/−*^ transgenic mice with muscles of sex and age-matched control mice. The major finding of this analysis is that deleting *Lats1*^*−/−*^ had 11% more type I fibers, 50% more *Myh7* mRNA, and 80% more type I myosin heavy chain when compared to matched wild-type controls. This suggests that Lats1 regulates fiber type proportion in mice. Furthermore, *Lats1* gene expression is upregulated in C2C12 myotubes treated with either the hypertrophy-stimulating agent clenbuterol or the energy stress-related drug AICAR. Moreover, re-analysis of published datasets revealed that *Lats1* expression increases after muscle injury and synergist ablation and that *LATS1* expression is higher in the muscles of young boys with DMD than in healthy muscles.

### *Lats1* deletion contributes to regulating muscle fiber type distribution in mouse skeletal muscle

The finding that *Lats1* deletion increased the proportion of slow, type I fibers fits the earlier discovery that a loss of *Vgll2* in mice reduced type I fibers when compared to wild-type mice (Honda et al. 2017). The fact that Lats1 is an inhibitor of Yap/Taz-Tead1-4 and that Vgll2 is a Tead1-4 coregulator suggest that a change of Tead1-4 activity in muscle can shift the fiber type composition of skeletal muscle. This adds the Hippo pathway to the list of pathways that regulate fiber type proportions. Other pathways in this list are the calcineurin-NFAT and ERK pathways (Ehlers et al. 2014; Schiaffino and Reggiani 2011). However, other Hippo gene mutations did not affect the distribution of muscle fiber types and it is unclear why some Hippo genes can have this effect whilst others have not.

### Regulation of *Lats1* expression in skeletal muscle

We then performed C2C12 cell culture experiments and reanalyzed published data sets to find out whether *Lats1* expression or phosphorylation is affected by exercise, damage, disease, or specific drugs. First, we experimentally investigated whether *Lats1* expression is changed in C2C12 myotubes hypertrophy-stimulated with clenbuterol or exposed to energy stress via AICAR. We found that AICAR increases *Lats1* expression by 1.3 fold. Up-regulation of *Lats1* in response to energy stress treatment with AICAR has been reported in different cell models such as in mouse embryonic fibroblasts and human retinal pigment epithelial-1 cells (Philippe et al. 2018). This suggests that *Lats1* expression responds to exercise-related stimuli that may affect muscle fiber type-specific gene expression. Second, our re-analysis of existing datasets revealed that *Lats1* expression is elevated in synergist ablation-loaded hypertrophying plantaris muscle (Lukjanenko et al. 2013). Additionally, *Lats1* expression is upregulated in regenerating muscles after cardiotoxin-induced injury (Chaillou et al. 2013) (Fig. 4 b–c). The reanalysis of published datasets also revealed that *LATS1* expression is 63% higher in the quadriceps of young boys with Duchenne muscular dystrophy compared to healthy quadriceps muscle (Fig. 4 a). Vita et al. 2018 also reported that LATS1/2 kinase activity increases in five different muscles (quadriceps, biceps, diaphragm, gastrocnemius, and EDL) in mdx mice that is a popular model for studying DMD. In DMD muscles, high miR-21 expression leads to an increase of Lats activity. Activation of both miR-21 and Lats1 can cause direct suppression of Yap activity and results in muscle degeneration and weakness (Vita et al. 2018). These data suggest that Lats1 is transcriptionally regulated in skeletal muscle.

The limitation of the study is that we only compared 3–5 mutant hindlimbs *(Lats1*^*−/−*^ (n = 4)*, Mst2*^*−/−*^ (n = 5)*, Vgll3*^*−/−*^ (n = 3)*,* and *Vgll4*^*+/−*^ (n = 5) to age and sex-matched wild-type controls. However, we additionally measured *Myh7* mRNA and myosin type I protein levels to confirm ourselves of the shift in fiber type.

In summary, *Lats1*^*−/−*^ mice had 11% more type I fibers, and that *Lats1*^*−/−*^*, Mst2*^*−/−*^*, Vgll3*^*−/−*^*, Vgll4*^*+/−*^ transgenic mice have no pathological skeletal muscle phenotype. Moreover, synergist ablation, muscle injury, and muscular dystrophy all increase the expression of *Lats1.*

## Supplementary Information

Below is the link to the electronic supplementary material.

Supplementary file1 (DOCX 32 kb)

Supplementary file2 (TIF 7618 kb)

Supplementary file3 (TIF 6929 kb)

Supplementary file4 (TIF 5426 kb)
