# Supplementary material for: Skeletal muscle phenotyping of Hippo gene-mutated mice reveals that Lats1 deletion increases the percentage of type I muscle fibers
Source: Transgenic Res. 2022 Jan 5;31(2):227–37. doi: 10.1007/s11248-021-00293-4 (PMC8993742; doi:10.1007/s11248-021-00293-4)
Supplement: Supplementary file 1 — Supplementary file1 (DOCX 32 kb) [file 11248_2021_293_MOESM1_ESM.docx]

**Skeletal muscle phenotyping of Hippo gene-mutated mice reveals that *Lats1* knock out increases the percentage of type 1 muscle fibers**

Fakhreddin Yaghoob Nezhad^1^ Anette Riermeier^1^ Martin Schönfelder^1^ Lore Becker^2^ Martin Hrabĕ de Angelis^2,3,4^ Henning Wackerhage^1^ (✉)

1) Exercise Biology Group, Technical University of Munich, Faculty of Sport and Health Sciences, Munich, Germany

2) Institute of Experimental Genetics, German Mouse Clinic, Helmholtz Zentrum München, Neuherberg, Germany

3) Chair of Experimental Genetics, TUM School of Life Sciences, Technische Universität München, Freising, Germany

4) German Center for Diabetes Research (DZD), Neuherberg, Germany

Corresponding author e-mail: [henning.wackerhage@tum.de](mailto:henning.wackerhage@tum.de)

# Supplementary Data

**Table S1** Compilation of publicly available IMPC data of muscle phenotypes

| Protein (*Gene*) | Gene mutation | IMPC size & muscle phenotypes | Muscle phenotypes (this study) |
| --- | --- | --- | --- |
| Mst1 *(Stk4)* | HOM Del | No size & muscle phenotypes phenotype | Not tested |
| Mst2 *(Stk3)* | HOM Del | No size & muscle phenotypes phenotype | No Muscle phenotype |
| Lats1 *(Lats1)* | HOM Del | Higher grip strength in female (33%)  Lighter absolute lean body mass in male (18.6%) | Higher fiber type l (11%) |
| Lats1 *(Lats1)* | HET Del | Reduction of grip strength in male (27%) | Not tested |
| Tead1 *(Tead1)* | HET Del | Higher total body fat mass in female (51.6%) | Not tested |
| Tead3 *(Tead3)* | HOM Del | No size & muscle phenotypes phenotype | Not tested |
| Vgll3 *(Vgll3)* | HOM Del | No size & muscle phenotypes phenotype | No Muscle phenotype |
| Vgll4 *(Vgll4)* | HET Del | No size & muscle phenotypes phenotype | No Muscle phenotype |

**Abbreviations:** HET Del, Heterozygous Deletion; HOM Del, Homozygous Deletion

**Fig S1** No pathological effects in muscle structure and enzyme activity following Knockout of Hippo-related genes in-vivo. (a) NADH-Tetrazolium Reductase (NADH-TR) staining of Tibialis anterior muscle cross-sections from control and Hippo related genes knockout mice (high oxidative capacity in dark blue, low oxidative capacity in light blue, and non-oxidative in white) (n = 3-5). (b) Hematoxylin and Eosin (H&E) staining of the soleus muscle cross-sections from control and Hippo-related genes knockout mice (Nuclei in dark blue, cytoplasm in red) (n = 3-5). Scale Bar = 50 μm

**Fig S2** *Mst2, Vgll3,* and *Vgll4* knockout do not affect muscle fiber type distribution and muscle or fiber cross-sectional area (CSA) in Hind limb muscles. ATPase stained soleus muscle section of (**a**) wild type or Mst2^-/-^*,* (**b**) wild type or *Vgll3^-/-^*, and (**c**) wild type or *Vgll4^+/-^* mice (Fibers type 1 stain dark, type 2, and 2a stain light) (n = 3-5). Scale Bar= 200 μm (whole muscle); 50 μm (higher resolution)

**Fig S3** Genotyping of *Lats1* knockout skeletal muscle. Representative images of skeletal muscle genotyping from 16-week-old control and Lats1^-/-^ mice (n = 4). The amplified DNA sequences were subjected to the agarose gel electrophoresis and the PCR products were visualized by observing ethidium bromide-stained gels under Ultra Violet illumination. The abbreviations, kb, Kilobase; bp, base pair
