# Supplementary figures and images for: Skeletal muscle phenotyping of Hippo gene-mutated mice reveals that Lats1 deletion increases the percentage of type I muscle fibers
Source: Transgenic Res. 2022 Jan 5;31(2):227–37. doi: 10.1007/s11248-021-00293-4 (PMC8993742; doi:10.1007/s11248-021-00293-4)

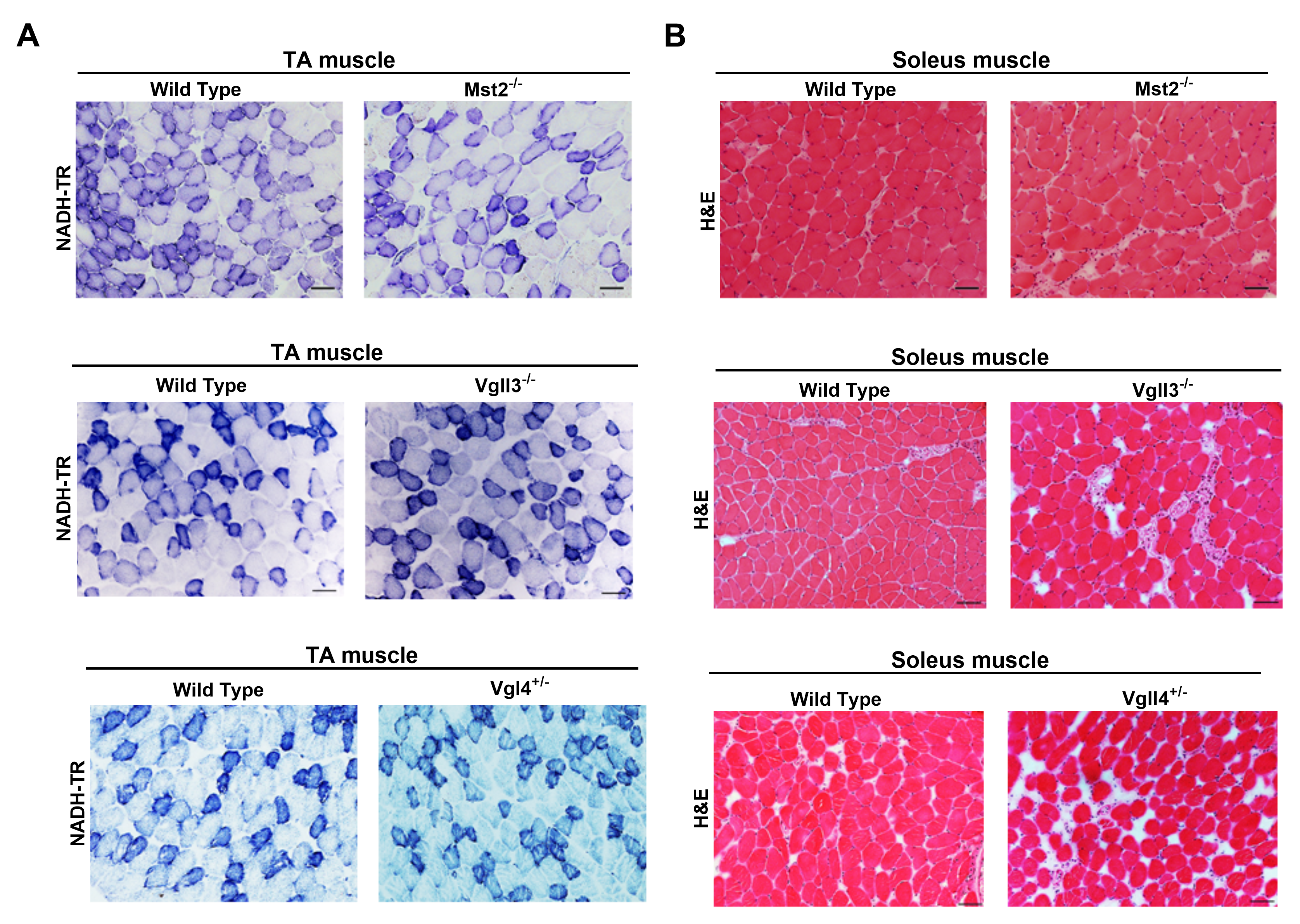

Supplement: Supplementary file 2 — Supplementary file2 (TIF 7618 kb) [file 11248_2021_293_MOESM2_ESM.tif]

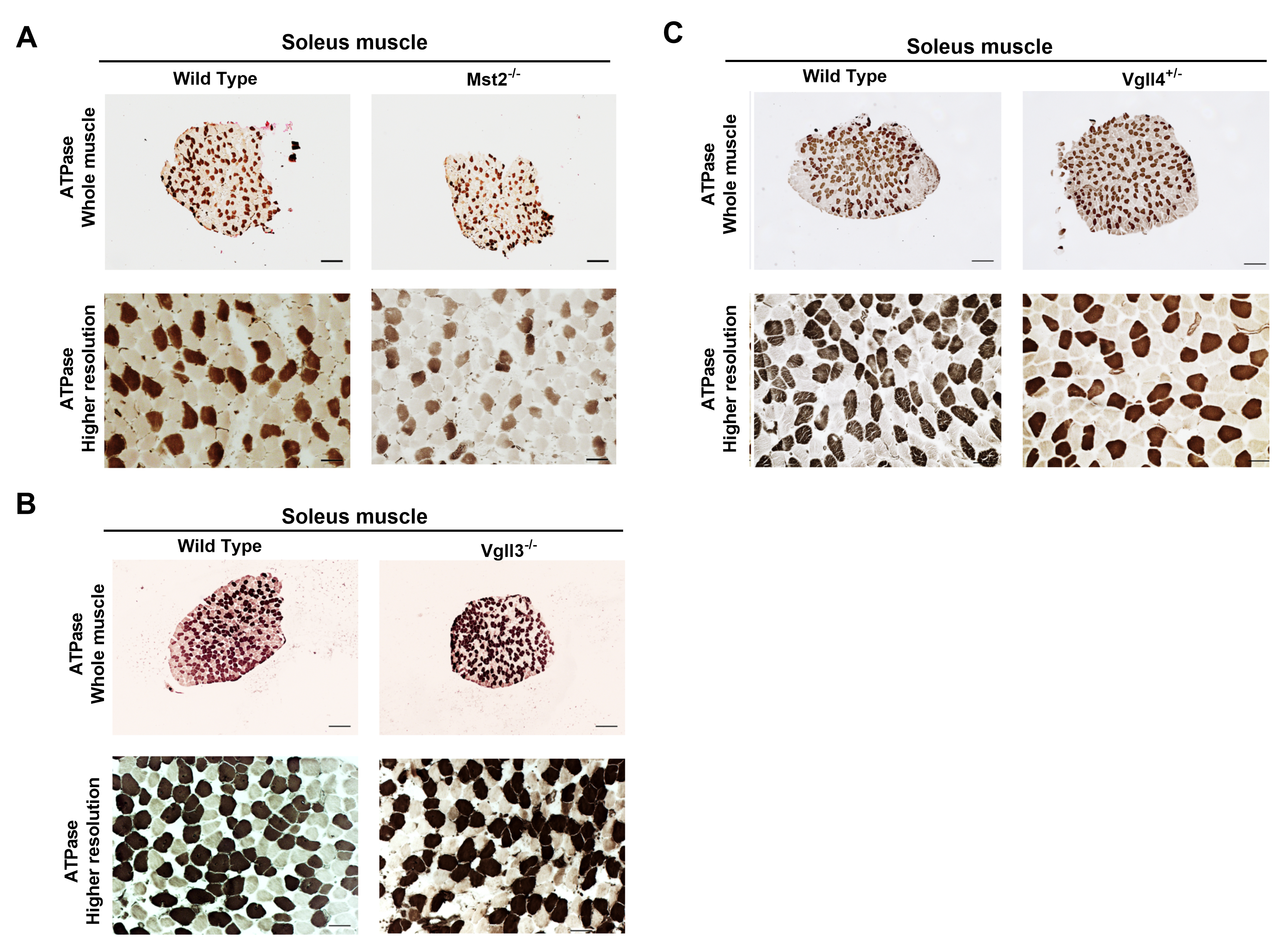

Supplement: Supplementary file 3 — Supplementary file3 (TIF 6929 kb) [file 11248_2021_293_MOESM3_ESM.tif]

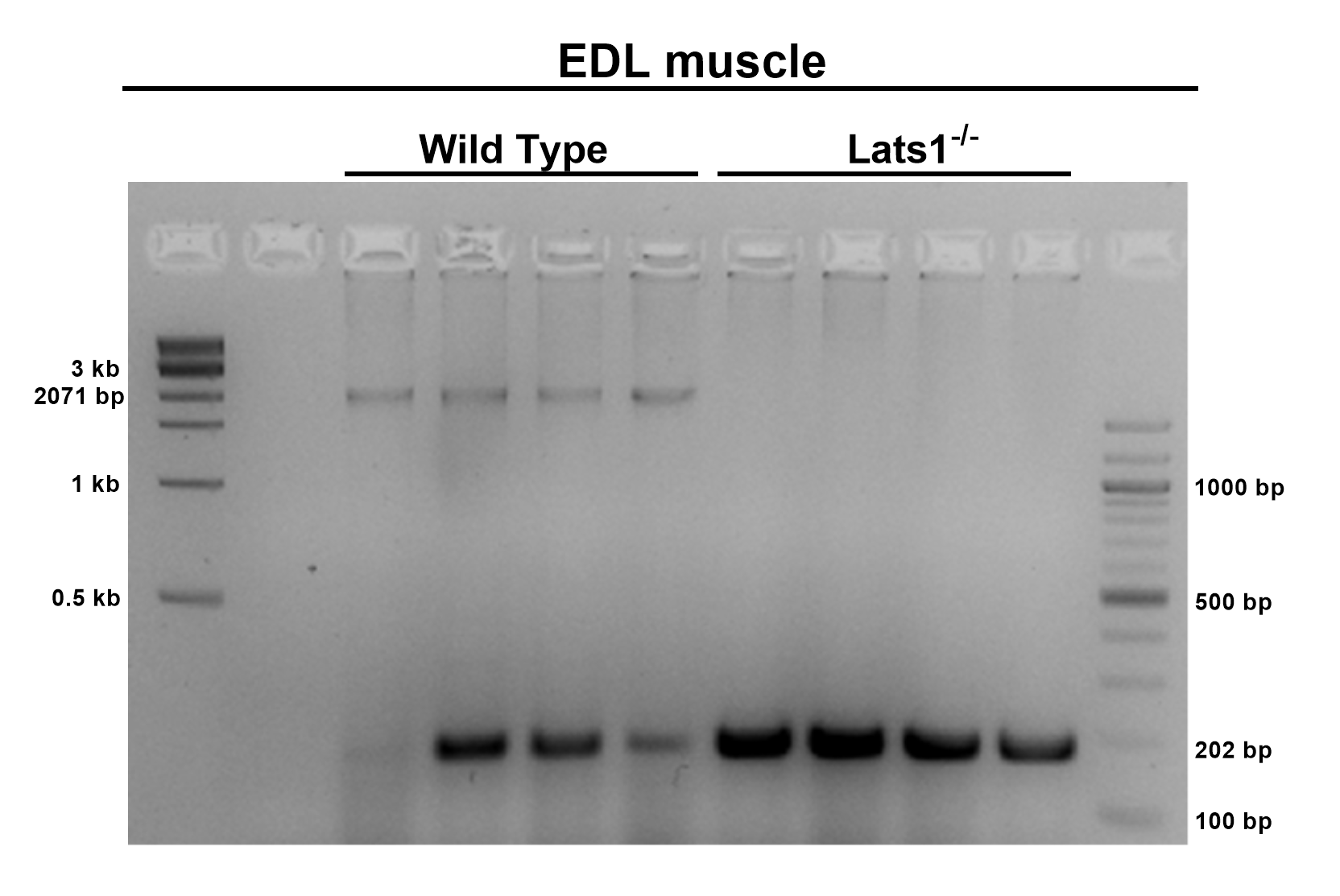

Supplement: Supplementary file 4 — Supplementary file4 (TIF 5426 kb) [file 11248_2021_293_MOESM4_ESM.tif]
